# The Outcomes of Pancreatic Transplantation from Pediatric Donors–A Single Institution Experience

**DOI:** 10.3390/jcm8091386

**Published:** 2019-09-04

**Authors:** Taihei Ito, Takashi Kenmochi, Naohiro Aida, Kei Kurihara, Akihiro Kawai, Atsushi Suzuki, Megumi Shibata, Izumi Hiratsuka, Midori Hasegawa

**Affiliations:** 1 Department of Transplantation and Regenerative Medicine, Fujita Health University, School of Medicine, Dengakugakubo 1-98, Kutsukakecho, Toyoake-shi, Aichi 470-1192, Japan; 2 Department of Endocrinology and Metabolism, Fujita Health University, School of Medicine, Dengakugakubo 1-98, Kutsukakecho, Toyoake-shi, Aichi 470-1192, Japan; 3 Department of Nephrology, Fujita Health University School of Medicine, Dengakugakubo 1-98, Kutsukakecho, Toyoake-shi, Aichi 470-1192, Japan

**Keywords:** pancreas transplantation, pediatric donor, brain-dead donor

## Abstract

Objectives: The aim of this study was to compare the outcomes of pancreatic transplantation from pediatric donors younger than 15 years of age to the outcomes of pancreatic transplantation from adult donors. Methods: Sixty patients underwent pancreatic transplantation in our facility from August 2012 to June 2019. These patients were divided into two groups according to the age of the donor: Cases in which the donor was younger than 15 years of age were classified into the PD group (*n* = 7), while those in which the donor was older than 15 years of age were classified into the AD group (*n* = 53). The outcomes of pancreas transplantation were retrospectively compared between the two groups. Results: Pancreatic graft survival did not differ between the PD and AD groups. Furthermore, there were no differences in the HbA1c and serum creatinine levels at three months, with good values maintained in both groups. The results of oral glucose tolerance tests (OGTTs) revealed that the blood glucose concentration did not differ between the two groups. However, the serum insulin concentration at 30 min after 75 g glucose loading was significantly higher in the PD group. Conclusion: The outcomes of pancreatic transplantation from pediatric donors may be comparable to those of pancreatic transplantation from adult donors and the insulin secretion ability after transplantation may be better.

## 1. Introduction

Since the revision of the organ transplantation law, pancreas transplantation from brain-dead donors has increased and 30–40 pancreas transplants are performed annually [1]. At the same time, organ donation from children under 15 years of age is also increasing. Among the 358 pancreas transplants performed in Japan by the end of 2018, 13 involved transplants from pediatric donors of <15 years of age. Seven of these procedures were performed in our facility.

Regarding pancreatic transplantation from small child donors, the following questions remain. Does the difficulty of vascular anastomosis increase and do such donors influence pancreatic graft survival due to associated postoperative complications, such as thrombosis? In addition, since the pancreatic graft volume is small, does size mismatch with adult recipients sometimes result in insufficient insulin secretion?

In the United States, *en bloc* grafting, including dual kidney and pancreatic grafts, has been successfully utilized for simultaneous pancreas and kidney transplantation from small pediatric donors [2,3,4]. However, the utilization of dual kidneys should be limited to cases involving very small donors because of a severe donor shortage in Japan and the difficultly associated with the use of *en bloc* grafts, including the pancreas and the use of the donor’s aorta for arterial anastomosis from the viewpoint of blood vessel sharing with liver transplantation.

In this study, we compared the outcomes of pancreatic transplantation from pediatric donors of less than 15 years of age to the outcomes of pancreatic transplantation from adult donors and clarified the usefulness of these procedures. All patients were treated in our facility.

## 2. Patients and Methods

### 2.1. Patients

All pancreatic transplant cases in Japan are registered in the Japan Organ Transplant Network. Patients waiting for transplantation are selected according to the following conditions, regardless of whether the cases involve pediatric or adult donors. Blood type compatibility and negativity on a direct crossmatch test are prerequisites for recipient selection. Priority for recipient selection is determined as follows: 1. The order of the recipients is arranged based on the number of HLA mismatches, with priority given to cases involving smaller numbers of HLA mismatches. 2. Cases are then prioritized in the order of simultaneous pancreas-kidney transplant (SPK), pancreas transplantation after kidney transplantation (PAK), pancreas transplantation alone (PTA). 3. Priority is given according to the length of the waiting period, with priority given to cases involving a longer waiting period. 4. Cases are prioritized in ascending order according to the estimated transport time, with priority given to cases with a shorter estimated transport time.

The criteria for accepting pediatric donor pancreas transplant in our facility were as follows: No history of diabetes, HbA1c ≤ 6.2%, and well-controlled blood glucose during ICU stay. In the case of SPK, the following conditions were also required: A normal serum creatinine level on admission or after sufficient fluid replacement and bodyweight ≥15 kg.

### 2.2. The Study Design

Sixty cases, in which pancreatic transplantation from brain-dead donors were performed at Fujita Medical University from August 2012 to June 2019, were divided into two groups according to the age of the donors. Cases involving donors of <15 years of age were classified into the pediatric donor group (PD group; *n* = 7), while those involving donors of >15 years of age were classified into the adult donor group (AD group; *n* = 53). The outcomes of pancreas transplantation were compared retrospectively.

The following items were compared as donor background factors: Age, sex, body weight, body mass index (BMI), cause of death, HbA1c, blood glucose, serum creatinine, LDH, Na, CRP, pancreatic graft weight, and total ischemic time of both the pancreas and kidney grafts. Pancreatic graft survival, as defined by a basal CPR level of >0.3 ng/mL, the insulin free rate at three months post-transplantation, the time course of the HbA1c and serum creatinine levels, and the 75 g-OGTT and glucagon tolerance test results at one month post-transplantation in the cases of graft survival were compared between the two groups as the outcomes of pancreatic transplantation.

### 2.3. Transplantation Methods and Immunosuppression Protocols

The transplantation methods applied in the PD group included SPK (*n* = 6) and PTA (*n* = 1). The transplantation methods applied in the AD group included SPK (*n* = 44), PAK (*n* = 7), and PTA (*n* = 2). All cases involved brain-dead donors. In all cases in which SPK was performed, it was performed with a single kidney graft. Both pancreatic and kidney grafts were transplanted from the same donor in all cases involving SPK recipients. Although blood vessel sharing is determined in consultation with the liver transplant team, most arterial reconstruction was anastomosed between a Carrel patch that included the roots of the celiac artery (CEA) and superior mesenteric artery (SMA) with the external iliac artery. In some cases, Y-graft anastomosis was required to anastomose the sphenopalatine artery (SPA) and SMA. The portal vein was extended as needed. Intestinal drainage was performed in all cases. In all cases involving pediatric or adult donors, vascular anastomosis for pancreatic transplantation was performed by the same surgeon, who was trained in microvascular techniques.

For induction therapy, basiliximab (20 mg/body) was administered on day 0 and 4 to all patients who underwent SPK, while anti-thymocyte globulin (1.5 mg/kg) was administered from day 0 to 4 to all patients who underwent either PAK or PTA. In all cases, tacrolimus (0.15 mg/kg [adjust to trough level: 3–8 ng/mL]), mycophenolate mofetil (1500 mg/body), and prednisolone (5 mg/body) were administered to maintain immunosuppression. During the perioperative period and the follow-up period after transplantation, all pancreatic transplantation recipients in both the PD and AD groups were managed by the same surgical team.

### 2.4. Statistical Analyses

All statistical analyses were performed using the EZR software program (freely distributed from the homepage of Saitama Medical Center Jichi Medical University), which extends the functionality of R and R commander [5]. The categorical variables were analyzed with an *x*^2^ test, the continuous variables were analyzed using the Mann-Whitney U-test. Kaplan-Meier curves and a log-rank test were used to analyze graft survival. *p* values of <0.05 were considered to indicate statistical significance.

### 2.5. Ethical Aspects

Before registration, all subjects gave their informed consent to the secretariat of islet transplants in Japan and information on the opt-out procedure was published on the Fujita Health University website (https://www.fujita-hu.ac.jp/). The study was conducted in accordance with the Declaration of Helsinki and the protocol was approved by the Ethics Committee of Fujita Health University (HM19-140).

## 3. Results

### 3.1. Background Factors and the Outcomes of Transplantation from Pediatric Donors

The backgrounds of the pediatric donors are summarized in Table 1. The youngest donor was 4 years of age, while the oldest was 11 years of age. The minimum weight was 18.5 kg. The pancreatic graft weight was 61–114 g. The donor’s HbA1c levels ranged from 4.7 to 5.5% and were within the normal range in all cases. The serum creatinine level was slightly high in Case 4 (0.82 mg/dl) but was normal in all other cases.

The backgrounds of the recipients and the results of transplantation are summarized in Table 2. PTA was performed in Case 2. In all other cases, simultaneous pancreas and kidney transplantation was performed. As arterial reconstruction, Cases 1 and 2 required Y-graft anastomosis. Carrel patches, which consisted of the celiac artery and superior mesenteric artery, were used in all other cases. Cases 1 and 2 also required portal vein prolongation. In Case 3, although no stenosis was found at the sites of portal vein anastomosis, blood flow stagnation between the splenic vein and the pancreatic graft was observed after reperfusion, and additional vein bypass was created by the placement of a vein graft between the splenic vein of the graft and the external iliac vein of the recipient.

In all cases, insulin withdrawal was achieved immediately after transplantation. In all cases of simultaneous pancreas and kidney transplantation, hemodialysis withdrawal was also achieved. However, acute rejection was observed in Cases 2 and 5. Despite treatment for rejection, Case 2 had a CPR level of <0.3 ng/mL at six months post-transplantation, leading to pancreatic graft loss. In all other cases, a good pancreatic and renal graft function was maintained.

### 3.2. Background Factors of the Pediatric Donor and Adult Donor Groups

Table 3 compares the backgrounds of the donors and recipients for the pediatric donor (PD) and adult donor (AD) groups. The donor body weight and body mass index (BMI) were significantly lower and the ICU stay was longer in the PD group. Laboratory analyses before procurement surgery revealed that the HbA1c, blood glucose, serum creatinine, and serum C-peptide (CRP) levels were significantly lower and the LDH level was significantly higher in the PD group. There were no differences in terms of the total ischemic time and the number of HLA-mismatches. However, the pancreatic graft weight was significantly lower in the PD group.

On the other hand, while the median HbA1c levels of the recipients were significantly higher before transplantation in the PD group, no differences in other background factors were observed between the two groups.

### 3.3. Pancreatic Graft Survival

**Figure 1:**
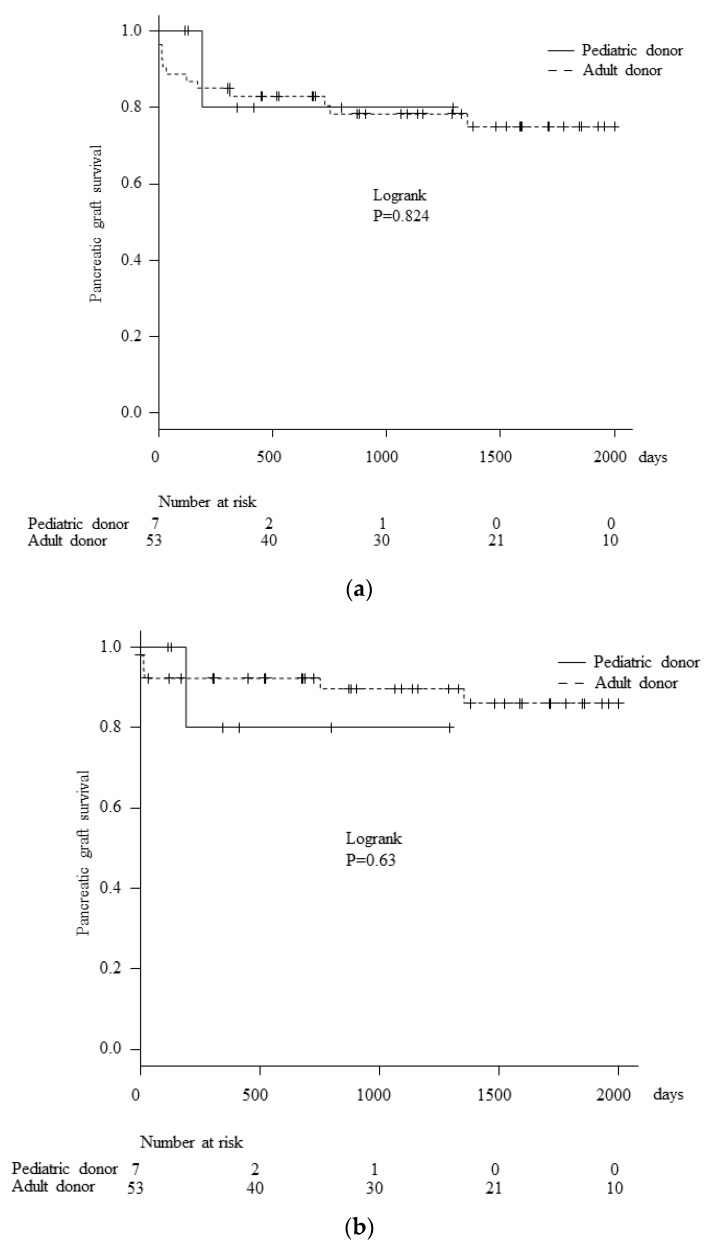
**Comparison of pancreatic graft survival.** There was no significant difference between the PD and AD groups in overall (**a**) or death-censored (**b**) pancreatic graft survival. Only one patient in the PD group experienced pancreatic graft loss due to rejection at six months post-transplantation. The graft function was well-maintained in the other cases.

Only one patient in the PD group experienced pancreatic graft loss due to rejection at six months post-transplantation, while in other cases, the graft function was well-maintained. On the other hand, 12 cases of pancreatic graft losses were experienced in the AD groups, including 10 cases of SPK and 2 cases of PTA. The causes of pancreatic graft loss in the AD groups were death with a functioning graft (*n* = 5 [myocardial infarction, *n* = 2; malignant neoplasm, *n* = 1; death due to accident, *n* = 1; multiple organ failure, *n* = 1), thrombosis (*n* = 4), rejection (*n* = 2), and other reasons (*n* = 1). In comparison to the AD group, there were no differences between the two groups in overall pancreatic graft survival (Figure 1a) or death-censored pancreatic graft survival (Figure 1b). With regard to the perioperative surgical complications, no cases of graft loss due to thrombosis were experienced in the pediatric donor group, while graft loss due to thrombosis occurred in 4 out of 53 cases (7.5%) in the adult donor group. Furthermore, perforation of the graft duodenum, which necessitated reoperation was observed in 4 of the 53 cases (7.5%) in the adult donor group, while there were no cases of perforation of the graft duodenum in the pediatric donor group.

**Figure 2:**
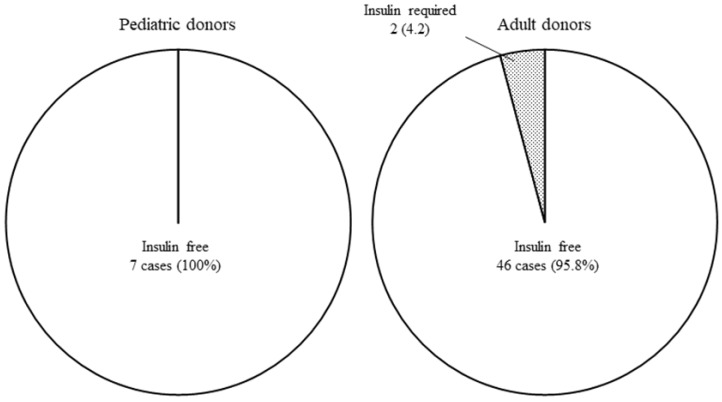
**The insulin free rate at three months post-transplantation.** Two cases in the AD group required insulin for glycemic control despite a positive CPR level (≥0.3 ng/ml), while all recipients in the PD group achieved an insulin free status.

The insulin free rate at three months after transplantation is shown in the Figure 2. Two cases in the AD group required insulin for glycemic control despite CPR positivity, while all recipients in the PD group achieved an insulin free status.

**Figure 3:**
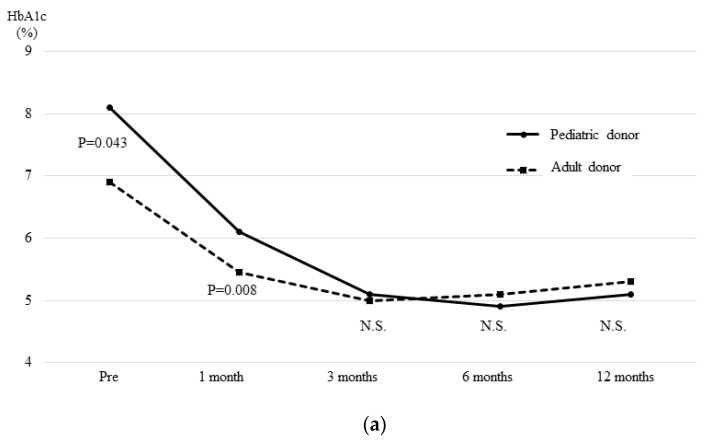

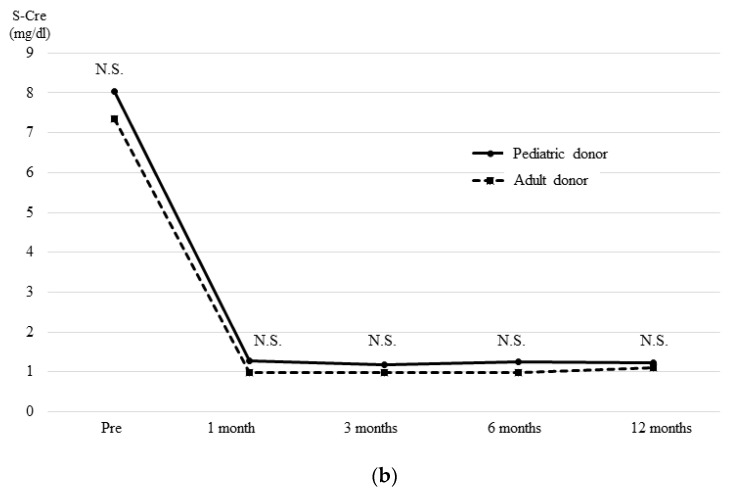
**The time course of the HbA1c (a) and serum creatinine (b) levels after transplantation.** The median preoperative HbA1c level of the recipients in the PD group was significantly higher than that in the AD group. The median HbA1c at one month post-transplantation was significantly higher in the PD group. However, there were no differences between the two groups after three months, with good values maintained in both groups. There were no differences between the two groups in the time course of the median serum creatinine level in cases in which simultaneous pancreas and kidney transplantation was performed, and a good renal graft function was maintained in all cases.

The time courses of the median HbA1c (Figure 3a) and serum creatinine level (Figure 3b) in the PD and AD groups are shown in Figure 3. The median preoperative HbA1c of recipients in the PD group was significantly higher than that in the AD group (PD group = 8.1% vs. AD group = 6.9, *p* = 0.043). Thus, the median HbA1c at one month post-transplantation was significantly higher in the PD group (PD group = 6.1 vs. AD group = 5.45, *p* = 0.008). However, there were no differences between the two groups after three months (PD group = 5.1 vs. AD group = 5.0, *p* = 0.874), with good values maintained in both groups.

No difference in the time course of the median serum creatinine level was observed between the two groups of patients who underwent simultaneous pancreas and kidney transplantation, and all patients maintained a good renal graft function.

### 3.4. The OGTT and Glucagon Stimulation Test Results at One Month after Transplantation

**Figure 4:**
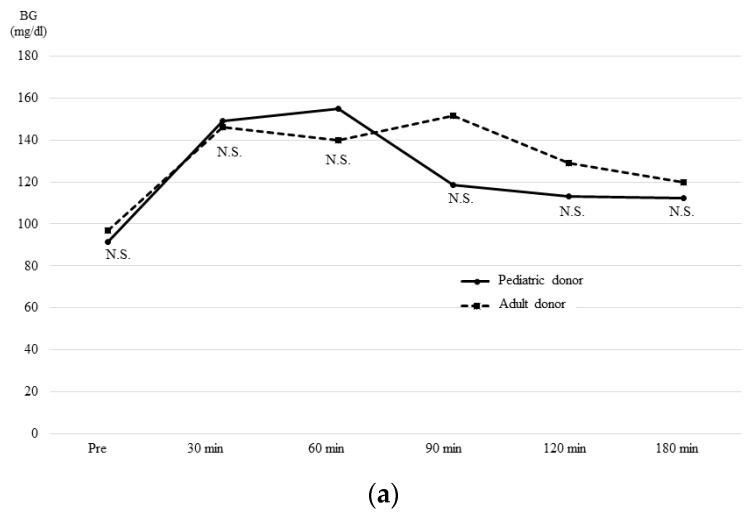

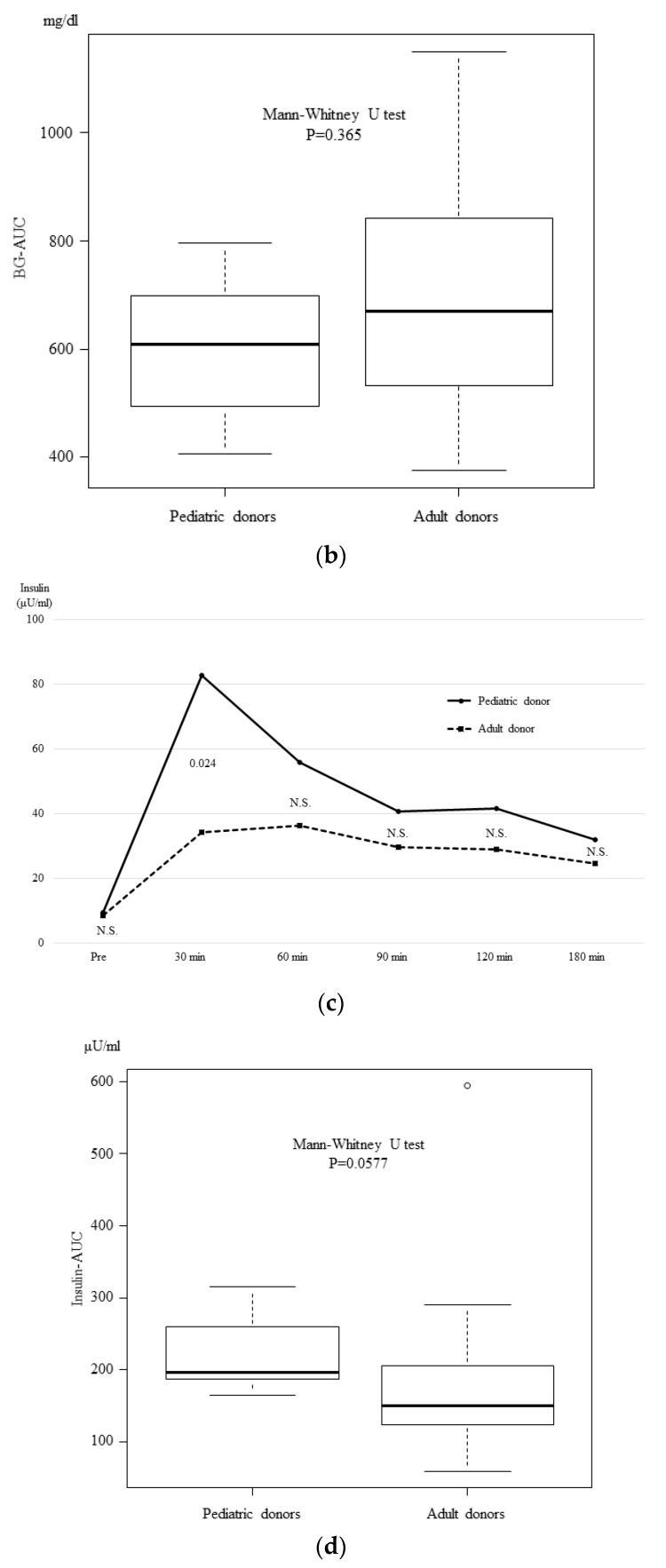
**The 75 g Oral glucose tolerance test (OGTT) results at one month post-transplantation.** The changes in blood glucose concentration (**a**) and the area under the curve (AUC) (**b**) did not differ between the two groups. However, the change in the serum insulin concentration (**c**) at 30 min after 75 g glucose loading was significantly higher in the PD group. As a result, although there was no significant difference, the AUC for insulin (**d**) tended to be higher in the PD group.

**Figure 5:**
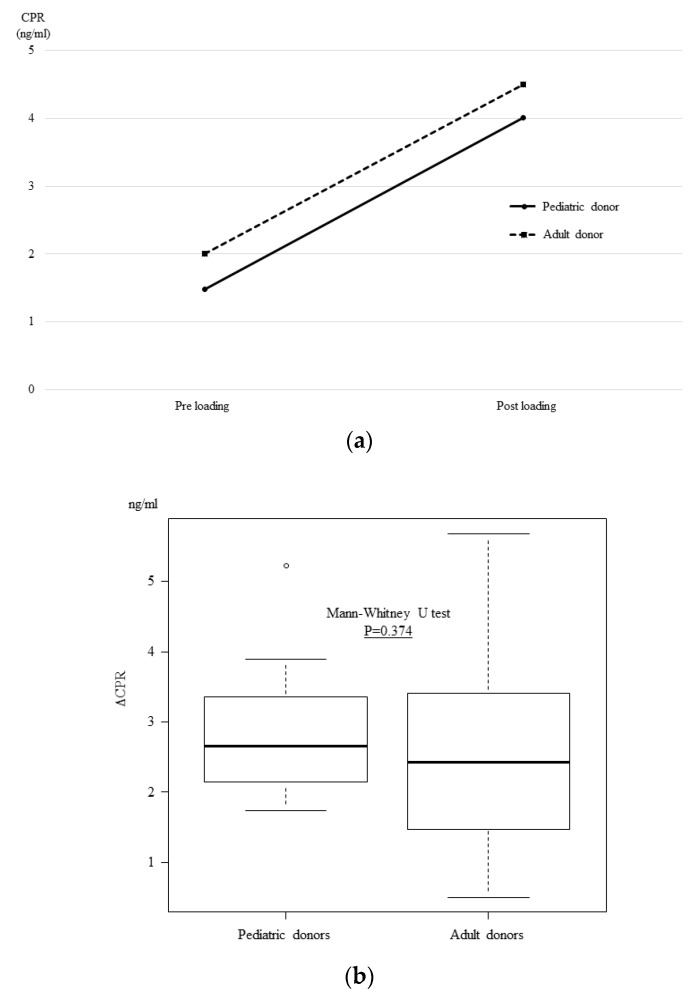
**The glucagon stimulation test results at one month after transplantation.** The glucagon stimulation test at one month post-transplantation showed that there was no significant difference between the two groups before or after glucagon loading (**a**) and the ΔCPR did not differ to a statistically significant extent (**b**).

A 75 g oral glucose tolerance test (OGTT) (Figure 4) and glucagon stimulation test (Figure 5) were performed at one month after transplantation. Regarding the OGTT results, changes in the blood glucose concentration (Figure 4a) and the area under the curve (AUC) (Figure 4b) did not differ between the two groups. However, the serum insulin concentration at 30 min after 75 g glucose loading was significantly higher in the PD group (Figure 4c) (PD group = 82.8μU/mL vs. AD group = 34.3 *p* = 0.024). As a result, although the difference was not statistically significant, the AUC for insulin tended to be higher in the PD group (Figure 4d) (PD group = 195.3μU/mL vs. AD group = 148.9 *p* = 0.0577).

On the other hand, in the glucagon stimulation test, no significant differences were observed between the two groups before or after glucagon loading (Figure 5a), or in the ΔCPR (Figure 5b) (PD group = 2.66 ng/mL vs. AD group = 2.43 *p* = 0.374).

## 4. Discussion

In the United States, pancreas transplantation from pediatric donors has been performed since the 1980s, and its usefulness has been reported. Nghiem DD et al. [6] reported seven cases of pancreatic transplantation from pediatric donors of 3–11 years of age. In these cases, the rates of thrombosis and early graft loss were 14% and 28%, respectively, while the rates in cases involving adult donors were 17.6% and 11.7%, respectively. They also indicated that the OGTT results at three months post-transplantation were similar to those of adult donors, but that the serum glucose level at 30 min after glucose loading was significantly higher in the pediatric group, despite the patients showing higher insulin secretion. These findings were somewhat different from the results that we experienced in procedures involving pediatric donors, as our patients showed better insulin secretion.

Since that time, there have been reports on pancreatic transplantation from pediatric donors from several institutions [2,3,4,7,8,9,10,11,12,13,14,15,16,17,18,19,20,21,22], and the results were comparable to those of adult donors. The youngest reported case (reported by Sageshima et al. [3]) involved a pediatric donor of 14 months of age; while the lowest donor body weight (reported by Nghiem et al. [6]) was 8.2 kg. Once the blood flow of the transplanted pancreatic graft can be secured, the smallness of the pancreas is unlikely to be a problem. The reason why there were no episodes of thrombosis or perforation of the graft duodenum in the PD group in our study, despite the difficulty of vascular anastomosis, was the relatively better tissue perfusion in the early period after transplantation. Aida et al. [23]. reported that early perioperative graft pancreatic tissue perfusion, as assessed by contrast-enhanced ultrasonography, tended to be better in younger donors. Better tissue perfusion is considered to be associated with faster the blood flow velocity in the splenic vein and portal vein in the pancreatic graft, and this is thought to reduce the risk of thrombus and to guarantee the blood flow of the graft duodenum.

One potential factor was the size of the anastomosed vessels and the distribution of kidney grafts, as 80% of pancreas transplants involve simultaneous pancreas and kidney transplantation. In the United States, Pelletier et al. [10]. and Kayler et al. [24,25]. reported that the outcomes of kidney transplantation from single kidney grafts from donors of <21 kg were significantly poor based on the Scientific Registry of Transplant Recipients (SRTR) data. Based on these results, a transplantation method using an *en bloc* graft including the bilateral kidneys and the pancreas was adopted in cases involving very small donors (e.g., donors of <21 kg). This *en bloc* graft also makes both arterial and vein anastomosis easier, as both the aorta and inferior vena cava are used as anastomotic orifices, which avoids narrow anastomosis.

The relatively small number of cases was one limitation of the present study. However, in Japan, even the number of cases of single kidney transplantation from brain-dead donors is limited, and it is unclear—as it is in the United States—whether the performance of single kidney transplantation from donors of ≤21 kg is associated with poor outcomes. In Japan, recipients are expected to weigh less than those in the United States. Thus, it is likely that the outcomes of single kidney transplantation from smaller pediatric donors would be better. Single kidney transplantation from small donors should be considered from the viewpoint of the donor shortage in Japan. However, a donor weight of ≥15 kg was required for SPK based on the consideration that single kidney transplantation from a donor of <15 kg would not provide a sufficient function for adult SPK recipients. Thus, dual kidney transplantation might be necessary. From this viewpoint, we consider that for kidney donation from a donor of <15 kg, priority should be given to age-matched pediatric kidney transplantation rather than adult SPK. The further accumulation of cases is expected in the future.

## 5. Conclusions

In conclusion, the outcomes of pancreatic transplantation from pediatric donors may be comparable to those pancreatic transplantation from adult donors, and the insulin secretion ability after transplantation may be better. The distribution of renal grafts remains a problem to be solved in the future, as >80% of pancreatic transplants involve simultaneous pancreas and kidney transplantation. It is necessary to properly consider the allocation according to the accumulation of kidney transplant results from pediatric donors and the numbers of donors in each country.

## Figures and Tables

**Table 1:** Donor characteristics.

Case	Age	Sex	Cause of Death	BW (kg)	HbA1c (%)	s-Cre (mg/dl)	Number of HLA Mismatches	TIT (Pancreas, min)	TIT (Kidney, min)	Graft Weight (Pancreas, g)
1	6	Male	Hypoxia	20.0	4.7	0.33	3	891	676	95
2	5	Female	Trauma	20.8	5.5	0.19	3	630	N/A	71
3	9	Male	Trauma	27.0	5.4	0.41	5	714	597	64
4	11	Female	Hypoxia	43.0	5.3	0.82	2	969	704	114
5	5	Male	Hypoxia	21.0	4.9	0.22	2	729	522	63
6	4	Female	Hypoxia	18.5	5.2	0.17	3	974	680	61
7	10	Female	CVA	29.1	5.4	0.47	2	801	606	75

BW, Body weight; CVA, Cerebrovascular accident; HLA, Human Leukocyte Antigen; s-Cre, serum creatinine; TIT, Total ischemic time.

**Table 2:** Recipient background and the results of transplantation.

Case	Recipient Age	Recipient Sex	Recipient BW (kg)	Operation Type	Arterial Reconstruction	Portal Vein Elongation	Episode of Rejection	Graft Survival	HbA1c *	s-Cre * (mg/dl)	Basal CRP ** (ng/mL)	CPR after Glucagon Load ** (ng/mL)	ΔCPR ** (ng/mL)
									(%)				
1	61	Male	61.3	SPK	Y-graft	+	-	Survive (43 M)	5.2	1.42	2.44	5.1	2.66
2	34	Male	55.1	PTA	Y-graft	+	+	Graft failure due to rejection (6 M)	5.3	N/A	1.19	4.01	2.82
3	36	Female	49.3	SPK	Carrel patch	SPV bypass	-	Survive (27 M)	5.1	0.76	2.44	7.66	5.22
4	50	Male	59.8	SPK	Carrel patch	-	-	Survive (14 M)	4.7	1.47	1.48	3.22	1.74
5	34	Female	56	SPK	Carrel patch	-	+	Survive (12 M)	5.1	0.94	1.3	3.46	2.16
6	56	Male	65.7	SPK	Carrel patch	-	-	Survive (5 M)	5	1.4	1.79	5.68	3.89
7	44	Female	51.9	SPK	Carrel patch	-	-	Survive (4 M)	4.2	0.91	1.43	3.57	2.14

BW, Body weight; CPR, C-peptide; s-Cre, serum creatinine; PTA, Pancreas transplantation alone; SPK, Simultaneous pancreas and kidney transplantation; SPV, Splenic vein. * at three months post-transplantation, ** at one month post-transplantation.

**Table 3:** The donor and recipient backgrounds of the pediatric donor (PD) group and the adult donor (AD) group.

		Group	Pediatric Donors	Adult Donors	*p* Value
		*n*	7	53	
Donor factors	Age		6 (4–11)	48 (17–67)	**<0.001**
	Sex	Male (%)	3 (42.9)	26 (49.1)	1
		Female (%)	4 (57.1)	27 (50.9)	
	BW (kg)		21.0 (18.5–43.0)	59.7 (40.0–94.1)	**<0.001**
	BMI (kg/m^2^)		16.0 (12.8–20.8)	22.2 (16.6–30.0)	**<0.001**
	Cause of death	CVA (%)	1 (14.3)	27 (52.9)	0.104
		Others (%)	6 (85.7)	24 (47.1)	
	ICU stay (days)		24 (7–35)	7 (2–34)	**0.005**
	Preoperative HbA1c (%)		5.3 (4.7–5.5)	5.5 (4.9–6.3)	**0.017**
	Preoperative BG (mg/dl)		97 (80–117)	128 (81–237)	**0.003**
	Preoperative s-Cre (mg/dl)		0.33 (0.17–0.82)	0.68 (0.23–6.93)	**0.004**
	Preoperative LDH (U/l)		1249 (871–2211)	688 (248–2323)	**0.002**
	Preoperative Na (mmol/l)		139 (130–143)	141 (114–166)	0.213
	Preoperative CRP (mg/dl)		7.43 (0.15–22.53)	17.51 (0.38–39.57)	**0.025**
	Pancreatic graft weight (g)		71 (61–114)	191 (95–352)	**<0.001**
	TIT (pancreas, min)		801 (630–974)	886 (494–1383)	0.189
	TIT (kidney, min)		641 (522–704)	706 (474–1124)	0.244
Recipient factors	Age		44 (34–61)	44 (31–62)	0.926
	Sex	Male (%)	4 (57.1)	17 (32.1)	0.226
		Female (%)	3 (42.9)	36 (67.9)	
	Preoperative HbA1c (%)		8.1 (6.3–12.3)	6.9 (4.9–9.8)	**0.043**
	Period of diabetic history (year)		29 (21–38)	29 (11–43)	0.926
	Period of hemodialysis history (year)		6.0 (1.5–11.0)	6.0 (0–20.0)	0.893

BMI, Body mass index; BW, Body weight; CVA, Cerebrovascular accident; HLA, Human Leukocyte Antigen; ICU, Intensive care unit; s-Cre, serum creatinine; TIT, Total ischemic time. Categorical variables were analyzed with the x^2^ test. Continuous variables were analyzed with the Mann-Whitney U-test.

## Abbreviations

AD: Adult donor; AUC, Area under the curve; BMI, Body mass index; BW, Body weight; CEA, Celiac artery; CPR, C-peptide; CVA, Cerebrovascular accident; HLA, Human Leukocyte Antigen; ICU, Intensive care unit; OGTT, Oral glucose tolerance test; PAK, Pancreas transplantation after kidney transplantation. PTA, Pancreas transplantation alone; PD, Pediatric donors; s-Cre, serum creatinine; SMA, Superior mesenteric artery; SPK, Simultaneous pancreas and kidney transplantation; SPV, Splenic vein; TIT, Total ischemic time.

## References

[1] Asaoka T., Ito T., Kenmochi T. (2018). The Japan Society for Pancreas and Islet Transplantation, The registry of Japanese pancreas and islet transplantation 2018. Ishoku.

[2] Buggenhout A., Hoang A.D., Hut F., Lekeufack J.B., Bali M.A., De Pauw L. (2004). Pediatric en bloc dual kidney-pancreas transplantation into an adult recipient: A simplified technique. Benefits of the en bloc kidney-pancreas transplantation technique in pediatric donors. Am. J. Transpl..

[3] Sageshima J., Ciancio G., Chen L., Selvaggi G., Nishida S., Akpinar E., Nesher E., Romano A., Misawa R., Burke G.W. (2010). Combined pancreas and en bloc kidney transplantation using a bladder patch technique from very small pediatric donors. Am. J. Transpl..

[4] Waldner M., Bachler T., Schadde E., Schiesser M., Immer F., Clavien P.A., Brockmann J.G. (2013). New surgical technique for pediatric en-bloc kidney and pancreas transplantation: The pancreas piggy-back. Transpl. Int..

[5] Kanda Y. (2013). Investigation of the freely available easy-to-use software ‘EZR’ for medical statistics. Bone Marrow Transpl..

[6] Nghiem D.D., Corry R.J., Cottington E.M. (1989). Function of simultaneous kidney and pancreas transplants from pediatric donors. Transplantation.

[7] Abouna G.M., Kumar M.S., Miller J.L., Rose L.I., Brezin J., Chvala R., Lyons P., Katz S.M., McSorley M. (1994). Combined kidney and pancreas transplantation from pediatric donors into adult diabetic recipients. Transpl. Proc..

[8] Van der Werf W.J., Odorico J., D’Alessandro A.M., Knechtle S., Becker Y., Collins B., Pirsch J., Hoffman R., Sollinger H.W. (1999). Utilization of pediatric donors for pancreas transplantation. Transpl. Proc..

[9] Rhein T., Metzner R., Uhlmann D., Serr F., Caca K., Weinert D., Hauss J., Witzigmann H. (2003). Pediatric donor organs for pancreas transplantation: An underutilized resource?. Transplant. Proc..

[10] Fernandez L.A., Turgeon N.A., Odorico J.S., Leverson G., Pirsch J.D., Becker B.N., Chin L.T., Becker Y.T., Knechtle S.J., Foley D.P. (2004). Superior long-term results of simultaneous pancreas-kidney transplantation from pediatric donors. Am. J. Transpl..

[11] Pelletier S.J., Guidinger M.K., Merion R.M., Englesbe M.J., Wolfe R.A., Magee J.C., Sollinger H.W. (2006). Recovery and utilization of deceased donor kidneys from small pediatric donors. Am. J. Transpl..

[12] Mazor R., Baden H.P. (2007). Trends in pediatric organ donation after cardiac death. Pediatrics.

[13] Illanes H.G., Quarin C.M., Maurette R., Sanchez N.G., Reniero L., Casadei D.H. (2009). Use of small donors (<28 kg) for pancreas transplantation. Transpl. Proc..

[14] Schenker P., Flecken M., Vonend O., Wunsch A., Traska T., Viebahn R. (2009). En bloc retroperitoneal pancreas-kidney transplantation with duodenoduodenostomy using pediatric organs. Transpl. Proc..

[15] Socci C., Orsenigo E., Santagostino I., Caumo A., Caldara R., Parolini D., Aldrighetti L., Castoldi R., Frasson M., Carvello M. (2010). Pancreata from pediatric donors restore insulin independence in adult insulin-dependent diabetes mellitus recipients. Transpl. Proc..

[16] Biglarnia A.R., Bennet W., Nilsson T., Larsson E., Magnusson A., Yamamoto S., Lorant T., Sedigh A., von Zur-Muhlen B., Backman L. (2014). Utilization of small pediatric donors including infants for pancreas and kidney transplantation: Exemplification of the surgical technique and the surveillance. Ann. Surg..

[17] Fisher R.A. (2014). Commentary on “Utilization of small pediatric donors including infants for pancreas and kidney transplantation”. Ann. Surg..

[18] Chiari D., Bissolati M., Gazzetta P.G., Guarneri G., Tomanin D., Maffi P., Secchi A., Rosati R., Socci C. (2016). Pancreas Transplantation from Very Small Pediatric Donor Using the “Cephalic Placement” Technique: A Case Report. Transpl. Proc..

[19] Spaggiari M., Bissing M., Campara M., Yeh C.C., Tzvetanov I., Jeon H., Benedetti E. (2017). Pancreas Transplantation from Pediatric Donors: A United Network for Organ Sharing Registry Analysis. Transplantation.

[20] Christensen K., Kennedy A., Kim R., Martinez E., Campsen J. (2018). Pancreatic Grafts from Pediatric Donors Do Not Appear to Grow After Transplantation into Adults. Cureus.

[21] Spaggiari M., Di Bella C., Di Cocco P., Campara M., Galen K., Gheza F., Oberholzer J., Benedetti E., Tzvetanov I. (2018). Pancreas Transplantation from Pediatric Donors: A Single-Center Experience. Transplantation.

[22] Dobbs S., Shapey I.M., Summers A., Moinuddin Z., van Dellen D., Augustine T. (2019). Simultaneous en-bloc pancreas and kidney transplantation from a small pediatric donor after circulatory death. Am. J. Transpl..

[23] Aida N., Kenmochi T., Ito T., Nishikawa T., Hiratsuka I., Shibata M., Suzuki A., Hasegawa M., Kawai A., Kusaka M. (2018). Prediction of Insulin Secretion Ability with Microcirculation Evaluated by Contrast-enhanced Ultrasonography in Pancreas Transplantation. Pancreas.

[24] Kayler L.K., Magliocca J., Fujita S., Kim R.D., Zendejas I., Hemming A.W., Howard R., Schold J.D. (2009). Recovery factors affecting utilization of small pediatric donor kidneys. Am. J. Transpl..

[25] Kayler L.K., Magliocca J., Kim R.D., Howard R., Schold J.D. (2009). Single kidney transplantation from young pediatric donors in the United States. Am. J. Transpl..

